# Identification of a novel long non-coding RNA within *RUNX1* intron 5

**DOI:** 10.1186/s40246-019-0219-1

**Published:** 2019-07-31

**Authors:** Nicolás Schnake, Marcela Hinojosa, Soraya Gutiérrez

**Affiliations:** Laboratory of Epigenetics [EpiGene], Departamento de Bioquímica y Biología Molecular, Facultad de Ciencias Biológicas, Universidad de Concepción, Barrio Universitario s/n, Concepción, Chile

**Keywords:** *RUNX1*, Intron, Breakpoint cluster region, Promoter, Non-coding RNA

## Abstract

**Background:**

*RUNX1* gene, a master regulator of the hematopoietic process, participates in pathological conditions as a partner for several genes in chromosomal translocations. One of the most frequent chromosomal translocations found in acute myeloid leukemia patients is the t(8;21), in which *RUNX1* and *ETO* genes recombine. In *RUNX1* gene, the DNA double-strand breaks that originate the t(8;21) are generated in the intron 5, specifically within three regions designated as BCR1, BCR2, and BCR3. To date, what determines that these regions are more susceptible to DNA double-strand breaks is not completely clear. In this report, we characterized *RUNX1* intron 5, by analyzing DNase-seq and ChIP-seq data, available in the ENCODE Project server, to evaluate DNaseI hypersensitivity and the presence of the epigenetic mark H3K4me3 in 124 and 51 cell types, respectively.

**Results:**

Our results show that intron 5 exhibits an epigenetic mark distribution similar to known promoter regions. Moreover, using the online tool YAPP and available CAGE data from the ENCODE Project server, we identified several putative transcription start sites within intron 5 in regions BCR2 and BCR3. Finally, available EST data was analyzed, identifying a novel uncharacterized long non-coding RNA, which is expressed in hematopoietic cell lines as shown by RT-PCR. Our data suggests that the core promoter of the novel long non-coding RNA locates within the region BCR3.

**Conclusion:**

We identified a novel long non-coding RNA within *RUNX1* intron 5, transcribed from a promoter located in the region BCR3, one of the chromosomal breakpoints of *RUNX1* gene.

**Electronic supplementary material:**

The online version of this article (10.1186/s40246-019-0219-1) contains supplementary material, which is available to authorized users.

## Background

Acute myeloid leukemia (AML) is a type of blood cancer that affects 0.6–11.0 in 100,000 people worldwide [1]. Depending on the cytogenetic characteristics of patients, the WHO distinguishes several AML types [2]. To date, a wide variety of chromosomal aberrations have been associated with AML: inversions, deletions, additions, and translocations [2]. One of the most frequent reciprocal translocations, found in 4–8.3% of AML patients, is the t(8;21) [3]. In this translocation, the genes *RUNX1* (*AML1*, *CBFA2*) and *ETO* (*RUNX1T1*, *MTG8*) recombine, generating a fusion protein that disrupts the hematopoietic process [4, 5]. *RUNX1* gene is the master regulator of definitive hematopoiesis in humans [6, 7]; therefore, defective cell differentiation contributes to the onset of leukemia.

For a chromosomal translocation to occur, it is necessary that a DNA double-strand break (DSB) takes place first. Despite the genomic size of *RUNX1* (260 kb) and *ETO* (148 kb) genes, it has been determined that the chromosomal breakpoints associated with the t(8;21) in both genes are found within specific introns: intron 5 in *RUNX1* gene and intron 1 in *ETO* gene [8]. In *RUNX1* intron 5 (24.8 kb), three smaller regions were identified as the chromosomal breakpoints involved in the translocation in AML patients and were named breakpoint cluster regions (BCRs): BCR1 (0.8 kb), BCR2 (4.2 kb), and BCR3 (2.1 kb) [9]. In hematopoietic cell lines, regions BCR2 and BCR3 presented DNaseI hypersensitivity and also harbored topoisomerase II cleavage sites [9], features that suggest chromatin accessibility and could be associated with a higher probability of DSB generation. Previous work in our lab showed that, along *RUNX1* intron 5, in HL-60 cells, there was an association of acetylated histone H3 [10]. Histone H3 tail acetylation is an epigenetic mark commonly associated with a more relaxed, accessible chromatin state [11]. Despite all this, to date, it is not completely clear what determines that the BCRs found within *RUNX1* intron 5 are more likely to suffer DSBs, as well as if this is an exclusive trait of *RUNX1* gene in hematopoietic cells.

In this work, our aim is to further characterize *RUNX1* intron 5 across different cell types, mainly through an in silico analysis of available DNase-seq, ChIP-seq, CAGE, and EST experimental data from the ENCODE Project server, comparing the presence of this features along *RUNX1* intron 5, with expected accessible and inaccessible genomic regions. Identifying and comparing the genomic features within *RUNX1* intron 5 will provide new insights regarding the chromosomal breakpoints in this gene, helping to understand what determines that these regions are more susceptible to DNA DSBs, and may be used as bases to analyze other genomic regions associated with chromosomal aberrations.

## Results

**Fig. 1:**
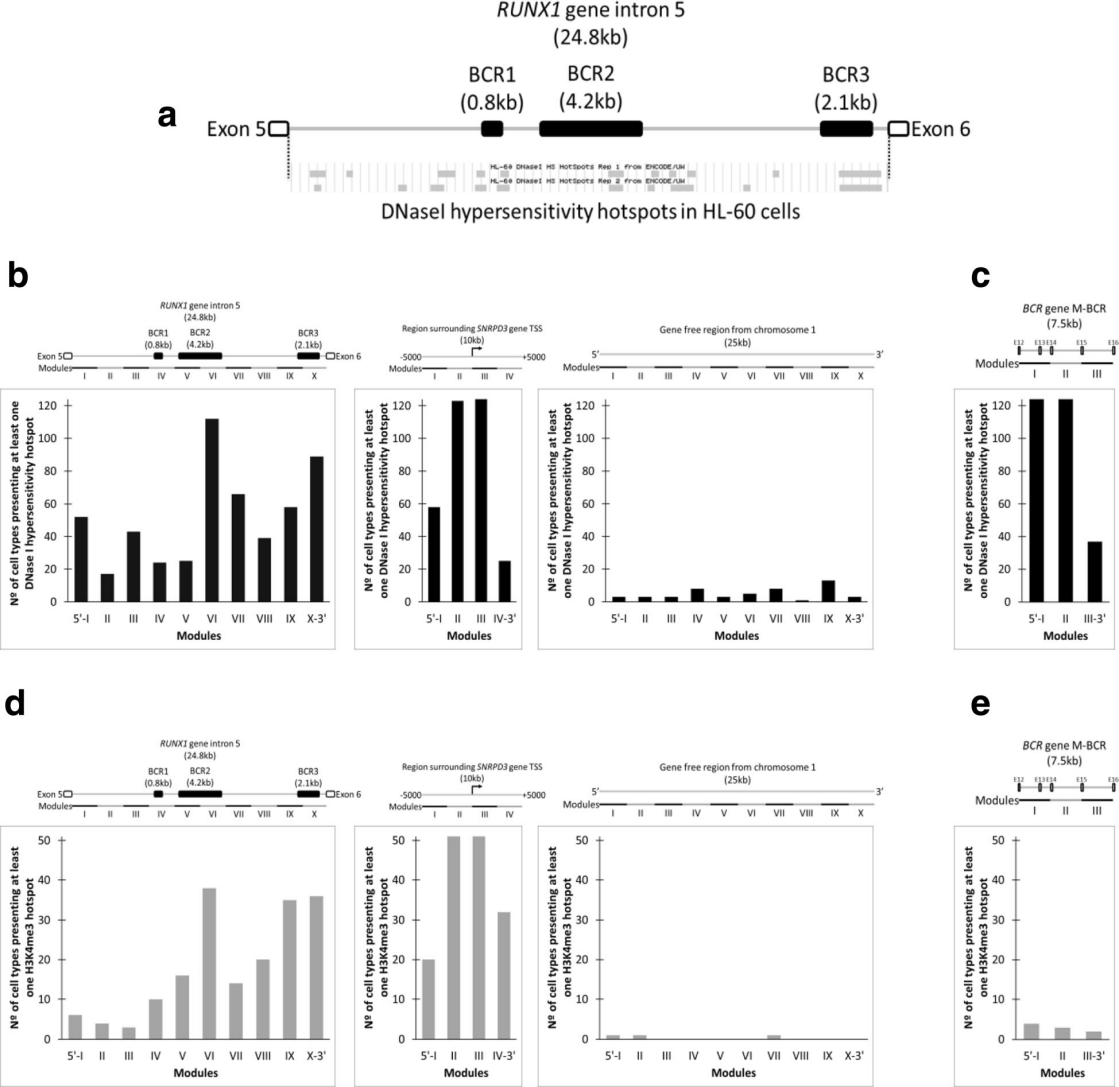
*RUNX1* intron 5 presents putative *cis*-regulatory elements that colocalize with chromosomal breakpoint regions. First, the presence of DNaseI hypersensitivity hotspots along *RUNX1* intron5 was analyzed using DNase-seq data of HL-60 cells available in the ENCODE Project server (**a**). Then, processed data from DNase-seq experiments performed on 124 cell types, available in the server ENCODE, was analyzed to evaluate DNaseI hypersensitivity along RUNX1 intron 5 (**b**, left panel), a region surrounding the transcription start site (TSS) of SNRPD3 gene (**b**, middle panel), a gene-free region found in chromosome 1 (**b**, right panel), and the major-BCR (M-BCR) from BCR gene (**c**). In the same way, processed data from ChIP-seq experiments performed in 51 cell types, available in the server ENCODE, was analyzed to evaluate the presence of the epigenetic mark H3K4me3 within RUNX1 intron 5 (**d**, left panel), a region surrounding the transcription start site (TSS) of SNRPD3 gene (**d**, middle panel), a gene-free region found in chromosome 1 (**d**, right panel), and the major-BCR (M-BCR) from BCR gene (**e**). Each region was subdivided into 2.5 kb-modules, as indicated by roman numerals. The bars represent the number of cell types that, at least, have one hotspot of DNaseI hypersensitivity (black) or the epigenetic mark H3K4me3 (gray), in each respective module

As it has been previously described, *RUNX1* intron 5 is involved in the generation of the chromosomal translocation (8;21) in AML patients; therefore, we expected to find an indication of chromatin accessibility along the region involved in the translocation in myeloid cells. To test this hypothesis, we analyzed the presence of DNaseI hypersensitivity hotspots along *RUNX1* intron 5 in HL-60 cells, using available DNase-seq data from the ENCODE Project server. As expected, we found DNaseI hypersensitivity hotspots along the region, some of them colocalizing with the previously described BCRs (Fig. 1a). In order to determine if chromatin accessibility within *RUNX1* intron 5 is a trait exclusively found in myeloid cells, we decided to further characterize *RUNX1* intron 5, evaluating the presence of DNaseI hypersensitivity along this region, using available DNase-seq hotspot data of 124 cell types from the ENCODE Project server. We found that most cell types presented several modules of DNaseI hypersensitivity along intron 5, which were identified as I through X from 5′ to 3′. Interestingly, modules VI and X, which colocalize with regions BCR2 and BCR3, presented DNaseI hypersensitivity in more than 80 different cell types (Fig. 1b, left panel). The distribution of DNaseI hypersensitivity in these modules is similar to what we found in the promoter of the reference gene *SNRPD3*, an expected accessible region (Fig. 1b, middle panel), and completely different from a gene-free region from chromosome 1 (Fig. 1b, right panel). To further compare *RUNX1* intron 5, we selected the well-known major-BCR region from the *BCR* gene, again finding the DNaseI hypersensitivity presence that resembles an accessible region (Fig. 1c). This indicates that chromosomal breakpoints localize within accessible genomic regions and that chromatin accessibility is not restricted to the cell type in which the genomic aberration t(8;21) is expected to occur in pathological conditions.

Considering the similarity of DNaseI hypersensitivity presence between the chromosomal breakpoint regions and the promoter region analyzed, we evaluated the presence of the epigenetic mark H3K4me3, found in promoters, using available H3K4me3 ChIP-seq hotspot data of 51 cell types from the ENCODE Project server. Surprisingly, in *RUNX1* intron 5, we found that in modules VI, IX, and X, which colocalize with regions BCR2 and BCR3, most cell types presented H3K4me3 (Fig. 1d, left panel). This result was similar to what was found in the promoter region of the gene *SNRPD3*, where the presence of the epigenetic mark was expected in all cell types (Fig. 1d, middle panel). In contrast, the gene-free region from chromosome 1 presented the epigenetic mark in only one cell type (Fig. 1d, right panel). Although in *RUNX1* intron 5 we found H3K4me3 colocalizing with chromosomal breakpoint regions, when we analyzed the major-BCR of *BCR* gene, only a few cell types presented the mark (Fig. 1e). These results show that the presence of H3K4me3 is not a common trait of chromosomal breakpoint regions and suggests that there might be promoters within *RUNX1* intron 5, in regions BCR2 and BCR3.

**Fig. 2:**
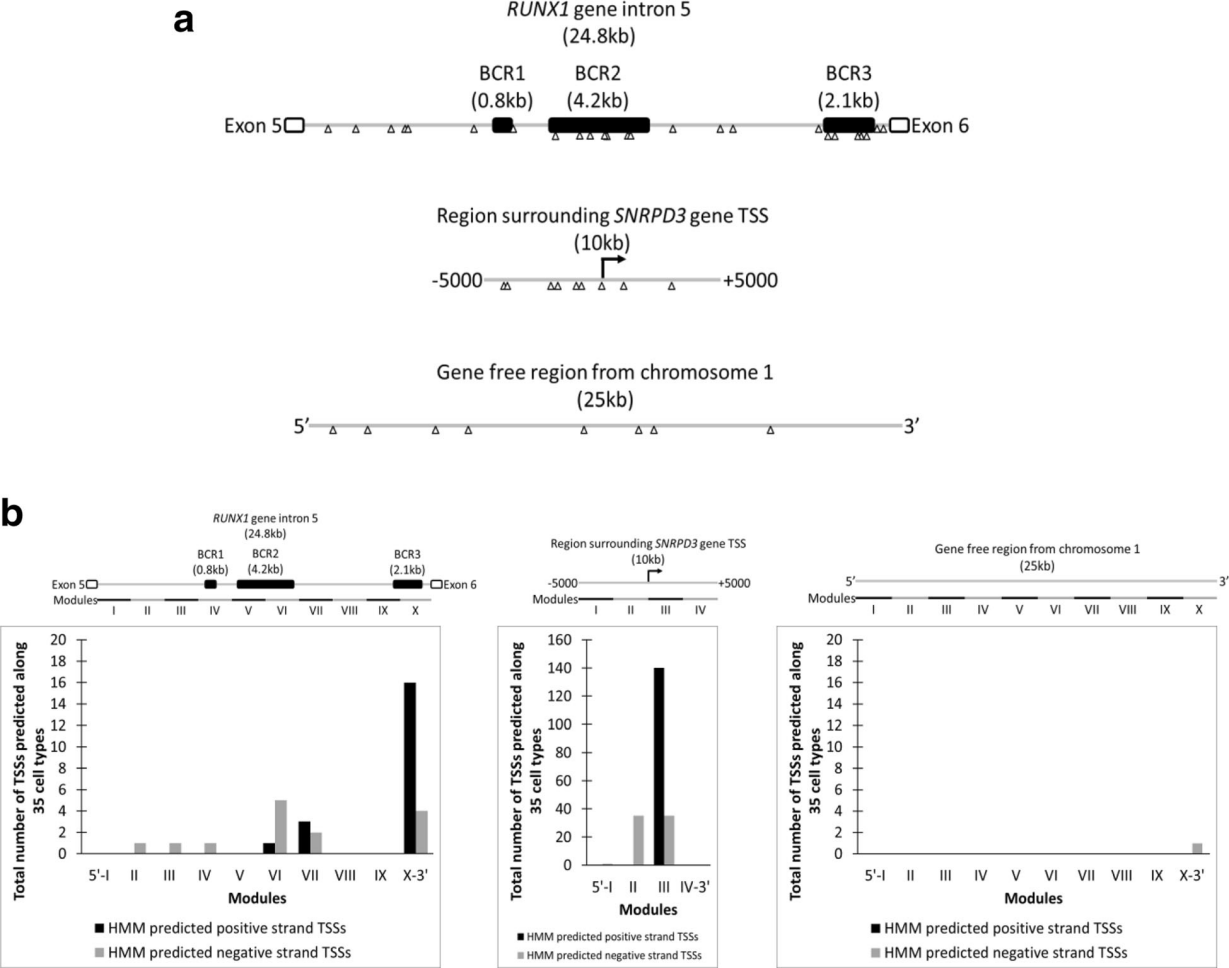
Multiple transcription start site predictions are found within chromosomal breakpoint regions of *RUNX1* intron 5. Using the free online software YAPP Eukaryotic Core Promoter Predictor, the presence of core promoter elements within RUNX1 gene intron 5 (**a**, top panel), a region surrounding the transcription start site (TSS) of SNRPD3 gene (**a**, middle panel), and a gene-free region found in chromosome 1 (**a**, bottom panel) was analyzed. The genomic sequence of the three regions was used as input. The cutoff score considered for this analysis was 0.99. Each arrow represents the central position of initiator elements (INR) found within each region, that is, potential TSSs. Additionally, processed data from CAGE experiments performed in 35 cell types, available in the server ENCODE, was analyzed to evaluate the presence of TSSs, predicted using Hidden Markov Models (HMM) based on raw CAGE data, within RUNX1 gene intron 5 (**b**, left panel); a region surrounding the TSS of SNRPD3 gene (**b**, middle panel); and a gene-free region found in chromosome 1 (**b**, right panel). Each region was subdivided into 2.5-kb modules, as indicated by roman numerals. The bars indicate the total number of TSSs predicted in each respective subdivision, distinguishing between TSSs in the positive strand (black) and TSSs in the negative strand (gray)

To investigate the possibility of intragenic promoters within *RUNX1* intron 5, we evaluated the distribution of predicted transcription start sites (TSSs) along *RUNX1* intron 5 using two strategies. In the first one, we performed an in silico prediction, using the online tool YAPP Eukaryotic Core Promoter Predictor. This tool identifies the sequences of core promoter elements within genomic regions, which indicate potential general transcription factor binding sites. In the second strategy, we evaluated the presence of TSSs predicted in 35 cell types using Hidden Markov Models (HMM) based on raw CAGE data, that is, data from actually expressed RNAs, available in the ENCODE Project server. For the in silico prediction, we considered only the initiator element (INR), which is located at the TSS of most genes, finding 25 INR in total along intron 5, with 7 of them colocalizing with the region BCR2 (4.2 kb), and 5 colocalizing with the region BCR3 (2.1 kb) (Fig. 2a, top panel). Comparatively, a 10-kb region surrounding the TSS of the reference gene *SNRPD3*, where at least 1 core promoter element was expected, has 9 INR (Fig. 2a, middle panel), and a 25-kb gene-free region from chromosome 1, matching the full size of *RUNX1* intron 5, has only 8 INR (Fig. 2a, bottom panel). On the other hand, using the HMM method, along *RUNX1* intron 5, we found 14 predictions of TSSs in the negative strand (in the same sense as *RUNX1* mRNA) and 20 predictions of TSSs in the positive strand (antisense to *RUNX1* mRNA). Interestingly, the module X, which contains 20 predictions in total, colocalizes with the region BCR3 (Fig. 2b, left panel). In comparison, in the 10-kb region around *SNRPD3* gene TSS, module III presented more than 100 TSS predictions across all cell types analyzed, matching the actual TSS location of that gene (Fig. 2b, middle panel), and for the gene-free region found in chromosome 1 (25 kb), there was only 1 TSS predicted (Fig. 2b, right panel). Therefore, the presence of predicted TSSs within *RUNX1* intron 5 is another indicator that suggests the presence of intragenic promoters in regions BCR2 and BCR3.

**Fig. 3:**
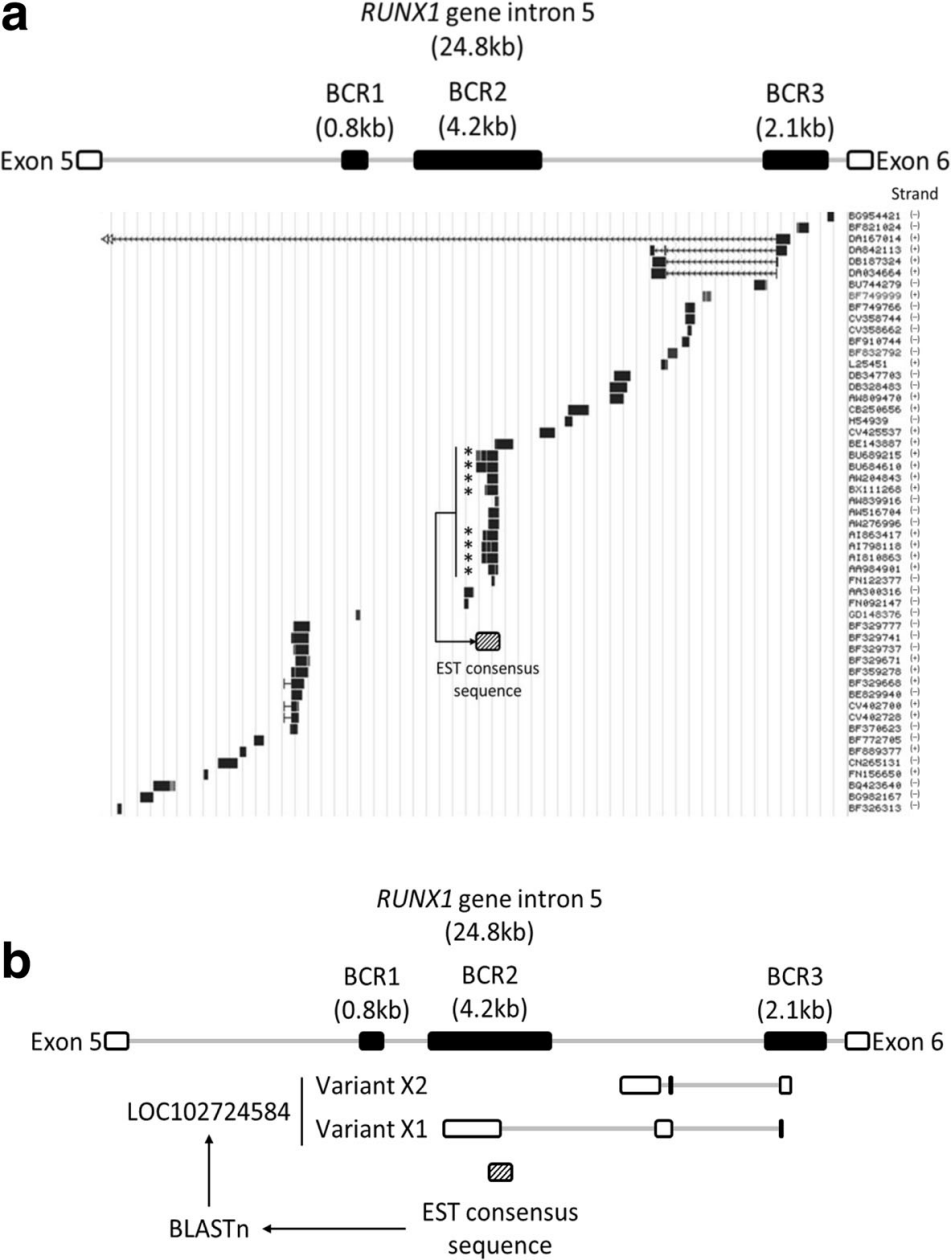
The promoter of different predicted uncharacterized long non-coding RNAs is located in one of the chromosomal breakpoint regions of *RUNX1* intron 5. Expressed sequence tagged (EST) data from different cell types, available in the server ENCODE, was analyzed to determine their distribution within RUNX1 intron 5. Several sequences were found, distinguishing between ESTs transcribed from the negative strand (in the same sense as RUXN1 mRNA) and ESTs transcribed from the positive strand (antisense to RUNX1 mRNA). Selected antisense ESTs, marked with an asterisk symbol, were used to perform a multiple alignment, in order to obtain a consensus sequence (dashed box) (**a**). This consensus sequence was used to perform a Nucleotide BLAST (nBLAST), finding predicted uncharacterized long non-coding RNAs, antisense to RUNX1 mRNA (**b**)

Taking into consideration the previous information, we wanted to identify RNA transcripts that could be originated in the regions BCR2 or BCR3. To this end, we analyzed expressed sequence tagged (EST) data, available from the ENCODE Project server. We found 53 ESTs in total spanning intron 5, 28 of them expressed from the negative strand (in the same sense as *RUNX1* mRNA) and 25 from the positive strand (antisense to *RUNX1* mRNA). Then, we selected a group of ESTs from the positive strand, colocalizing with the region BCR2, and performed a multiple alignment, in order to obtain a consensus sequence (Fig. 3a). This EST consensus sequence was used as input in the Nucleotide BLAST online tool. We found similarity with a predicted uncharacterized long non-coding RNA, which had its core promoter within the region BCR3, and was antisense to *RUNX1* mRNA (Fig. 3b). These features distinguish the sequence found from other long non-coding RNAs previously described in *RUNX1* gene *locus*, namely *RUNX1-IT1* [12] and *RUNXOR* [13], which are transcribed in the same sense as *RUNX1* mRNA, and, as for their promoters, one locates within intron 1 (*RUNX1-IT1*), and the other locates upstream from the TSS of *RUNX1* gene (*RUNXOR*). Thus, taken together, our results suggest that the promoter identified within the region BCR3 is functional.

**Fig. 4:**
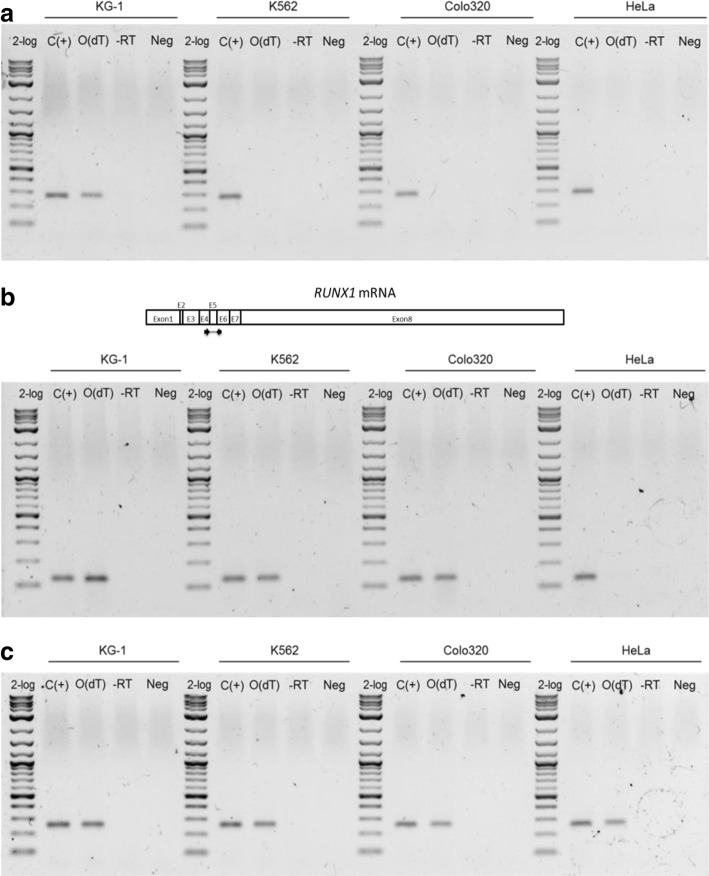
A novel long non-coding RNA was detected in hematopoietic cell lines. RT-PCR was performed to evaluate the expression of the previously identified long non-coding RNA in four cell lines, two hematopoietic (KG-1 and K562) and two non-hematopoietic (Colo320 and HeLa) (**a**). RUNX1 gene mRNA expression was evaluated in the same way (**b**, bottom panel) and also the expression of the reference gene SNRPD3 mRNA (**c**). A representation of RUNX1 mRNA is included, indicating the position of the primers used, flanking intron 5 (**b**, top panel). C(+), positive control; O(dT), cDNA synthesized using oligo(dT) as template; -RT. minus RT control; Neg, negative control (water as a template)

To detect this uncharacterized long non-coding RNA, we performed RT-PCR in four cell lines: KG-1, K562, Colo320, and HeLa. As shown in Fig. 4a, we only detected the sequence in KG-1 cells. In order to rule out that the detected sequence was a novel *RUNX1* isoform partially retaining intron 5, we performed a PCR with primers in exons flanking intron 5 (Fig. 4b, top panel), detecting a unique product of the size predicted if intron 5 was completely spliced. As expected, *RUNX1* mRNA was detected in three of the four cell lines: KG-1, K562, and Colo320 (Fig. 4b, bottom panel). Finally, as an expression control, we amplified a fragment of the reference gene *SNRPD3* mRNA (Fig. 4c). Taken together, these results suggest that the RNA molecule detected is not an isoform of *RUNX1*, but a novel intragenic long non-coding RNA, originated in a promoter located in the chromosomal breakpoint region of *RUNX1*, BCR3.

## Discussion

*RUNX1* is a gene that, in pathological conditions, participates in several chromosomal translocations associated with leukemia. To name a few, in a 2009 review by De Braekeleer et al. [14], t(3;21), t(8;21), t(12;21), and t(16;21) were described among the most frequent translocations found in AML patients, and more have been identified since [15–18]. As of date, what determines that this gene is prone to DSBs that originate translocations in myeloid cells is not fully understood. However, it has been described that for each translocation, the chromosomal breakpoint within *RUNX1* gene is located in specific introns, most notably introns 5 and 6 [19].

In this work, we studied *RUNX1* intron 5, a region associated with t(8;21), the most frequent *RUNX1* translocation in AML patients [3]. Within *RUNX1* intron 5, three BCRs were identified in 2002 by Zhang et al. [9]: BCR1, BCR2, and BCR3. Of the three, the region BCR3 presented DNaseI hypersensitivity in myeloid cells, a genomic feature that suggested that chromatin in that region was more accessible. In this case, we found that *RUNX1* intron 5 BCR3 presented promoter features (Figs. 1 and 2) and that this putative intragenic promoter was the origin of a novel uncharacterized long non-coding RNA (lncRNA), antisense to *RUNX1* mRNA (Figs. 3 and 4).

Our findings concur with what is known as nested genes, non-coding RNA (ncRNA) genes that are embedded to protein-coding host genes [20]. These ncRNAs can be either sense or antisense with respect to their host genes, and their promoters are often located within an intron [21]. It has been described that up to 50% of human genes possess at least one embedded ncRNA, including but not restricted to small nucleolar RNAs (snoRNAs), microRNAs (miRNAs), and lncRNAs. Around 50% of all snoRNAs and miRNAs are embedded to host genes; most of them are transcribed in the same sense as their host mRNA, and their expression is usually coordinated [20]. In fact, snoRNAs are long known to be spliced from the pre-mRNA of their host genes [22]. On the other hand, about 40% of all known lncRNAs are nested to a host gene [23], and the vast majority (80%) of them are antisense with respect to their host gene mRNA [20]. The promoters of these lncRNAs are often located within an intron [21]; their TSSs present epigenetic marks similar to protein-coding genes, including H3K4me3 [23]; and unlike snoRNAs and miRNAs, the expression of nested lncRNAs is usually not correlated with the expression of their host genes [24], which is what we found in this work.

Over the last decade, the role of lncRNAs has been described in different processes and diseases, including normal hematopoiesis [25–27] and leukemia [13, 28–30]. In this case, it seems that the transcription of the novel nested lncRNA detected in myeloid KG-1 cells (Fig. 4) results in a more relaxed chromatin organization at its promoter, located in the BCR3. Therefore, it is possible that this feature acts as a determinant of a higher probability of DSB generation in myeloid cells. It is necessary to address these interrogants experimentally to test that possibility. We recognize as well the importance of performing a complete characterization of this novel nested lncRNA and determine its function.

## Conclusion

In conclusion, through in silico analyses performed to determine the presence of genomic features (DNaseI hypersensitivity, the epigenetic mark H3K4me4, core promoter elements, TSSs predictions), and an EST analysis, along *RUNX1* intron 5, we have identified a novel nested lncRNA transcribed from a putative promoter located in the region BCR3 in *RUNX1* intron 5, one of the chromosomal breakpoints of *RUNX1* involved in the generation of the t(8;21).

## Methods

### DNase-seq and ChIP-seq data

DNase-seq and ChIP-seq data, available in the ENCODE Project server (https://genome.ucsc.edu/encode/dataMatrix/encodeDataMatrixHuman.html), was used to evaluate DNaseI hypersensitivity and the presence of the epigenetic mark H4K4me3 in four genomic regions: (1) *RUNX1* gene intron 5, (2) a region surrounding the transcription start site (TSS) of *SNRPD3* gene, (3) a gene-free region found within chromosome 1, and (4) the major-BCR (M-BCR) of *BCR* gene. The coordinates of these regions were obtained from the ENSEMBL Project (http://grch37.ensembl.org/index.html) (Additional file 1: Table S1), considering the human genome version hg19 to match the version used in ENCODE, and each region was subdivided into 2.5-kb modules. In total, 124 cell types that had DNase-seq data were considered (Additional file 2: Table S2), and from those, 51 cell types that also had H3K4me3 ChIP-seq data (Additional file 3: Table S3). The presence of DNaseI hypersensitivity and H3K4me3 hotspots in each module was evaluated visually, using the UCSC Genome Browser (https://genome.ucsc.edu/cgi-bin/hgTracks) and counting the number of cell types that, at least, had one DNase I or H3K4me3 hotspot signal, respectively.

### Core promoter element identification

Core promoter elements were identified using the online tool YAPP Eukaryotic Core Promoter Predictor (http://www.bioinformatics.org/yapp/cgi-bin/yapp.cgi) in three regions: (1) *RUNX1* gene intron 5, (2) a region surrounding the transcription start site (TSS) of *SNRPD3* gene, and (3) a gene-free region found within chromosome 1. The coordinates of these regions were obtained from the ENSEMBL Project (http://grch37.ensembl.org/index.html) (Additional file 1: Table S1) and the sequences from the NCBI website (https://www.ncbi.nlm.nih.gov). The three sequences were submitted as input, considering a cutoff score of 0.99.

### CAGE data

Processed data from CAGE experiments, available in the ENCODE Project server (https://genome.ucsc.edu/encode/dataMatrix/encodeDataMatrixHuman.html), was analyzed to evaluate the presence of transcription start sites (TSSs) predicted using Hidden Markov Models (HMM) based on raw CAGE data in three regions: (1) *RUNX1* gene intron 5, (2) a region surrounding the transcription start site (TSS) of *SNRPD3* gene, and (3) a gene-free region found within chromosome 1. The coordinates of these regions were obtained from the ENSEMBL Project (http://grch37.ensembl.org/index.html) (Additional file 1: Table S1), considering the human genome version hg19 to match the version used in ENCODE, and each region was subdivided into 2.5-kb modules. All 35 cell types that had CAGE data were considered, and the presence of TSSs in each module was evaluated visually, using the UCSC Genome Browser (https://genome.ucsc.edu/cgi-bin/hgTracks), counting the total number of TSSs along all cell types, and classifying them according to their transcription sense.

### Expressed sequence tagged data

Poly(A)+ EST data, available in the ENCODE Project server (https://genome.ucsc.edu/cgi-bin/hgTracks), was analyzed to identify ESTs within *RUNX1* gene intron 5. Genomic coordinates of this region were obtained from the ENSEMBL Project (http://grch37.ensembl.org/index.html) (Additional file 1: Table S1), considering the human genome version hg19 to match the version used in ENCODE. The 53 ESTs found were classified according to their transcription sense (e.g., in the same sense as or antisense to *RUNX1* mRNA) (Additional file 4: Table S4). Then, the following antisense sequences were selected: BU689215, BU684610, AW204843, BX111268, AI863417, AI798118, AI810863, and AA984901 (Additional file 4: Table S4), and with them, a multiple alignment was performed in order to obtain a consensus sequence. The consensus sequence was used as input in the online tool Nucleotide BLAST (https://blast.ncbi.nlm.nih.gov/Blast.cgi?PROGRAM=blastn&PAGE_TYPE=BlastSearch&LINK_LOC=blasthome), in order to identify full RNA predictions.

### Cell culture

KG-1 cells were cultured in IMDM medium, supplemented with 10% FBS, 2 mM l-glutamine, and 1% penicillin-streptomycin; K562 and Colo320 cells were cultured in RPMI 1640 medium, supplemented with 10% FBS, 2 mM l-glutamine, and 1% penicillin-streptomycin; HeLa cells were cultured in DMEM medium, supplemented with 10% FBS, 2 mM l-glutamine, and 1% penicillin-streptomycin. All cells were maintained at 37 °C, with 5% CO_2_.

### RNA detection

RT-PCR was performed to evaluate RNA expression in the different cell lines, using primer sets to detect *RUNX1* and *SNRPD3* mRNA, and another primer set to detect the novel predicted uncharacterized non-coding RNA. First, total RNA was extracted using the PureLink™ RNA mini kit (Ambion) and quantified using the Qubit® RNA BR Assay kit (Invitrogen) in a Qubit® 2.0 fluorometer. Then, 4 μg of total RNA was converted to cDNA using the SuperScript® III First-Strand Synthesis System (Invitrogen), as indicated by the manufacturer. Each reaction was diluted 1:25, and 1 μL was used to perform PCR, with the following program: [30 s at 95 °C–30 s at 58 °C–30 s at 72 °C] for 28 cycles. The three primer pairs used were [ncRNA F: AGC TCG CTG TCC TGT TCA TT; R: AGC TAG CAG GGC CAG ACA TA], [*RUNX1* F: GTC GAA GTG GAA GAG GGA AA; R: CCG ATG TCT TCG AGG TTC TC], and [*SNRPD3* F: TCT TCC TGC CAA GAT GTC TA; R: TAA CAT GGG TGC GTT CTT C]. Finally, PCR products were resolved using electrophoresis in 1% agarose gels, dyed with SYBR® Safe DNA Gel Stain (Invitrogen), a fluorophore that allows visualization under UV light.

## Additional files

Additional file 1: **Table S1.** Genomic coordinates of the analyzed regions. (XLSX 11 kb)

Additional file 2: **Table S2.** Presence of DNaseI hypersensitivity along *RUNX1* intron 5 and control regions, in 124 cell types. (XLSX 23 kb)

Additional file 3: **Table S3.** Presence of the epigenetic mark H3K4me3 along *RUNX1* intron 5 and control regions, in 51 cell types. (XLSX 15 kb)

Additional file 4: **Table S4.** EST sequences found in *RUNX1* intron 5. (XLSX 12 kb)

## Data Availability

The datasets supporting the conclusions of this article are included within the article, its additional files, and are publicly available in the ENCODE Project server human data matrix website [https://genome.ucsc.edu/encode/dataMatrix/encodeDataMatrixHuman.html].
